# Synthesis and characterization of semi-aromatic polyamides containing heterocyclic 1,3,5 s-triazine and methylene spacer group for thermally stable and colloidal property

**DOI:** 10.1080/15685551.2020.1795435

**Published:** 2020-07-26

**Authors:** A. B. Tamboli, B. S. Kalshetti, S. D. Ghodke, A. V. Diwate, N. N. Maldar

**Affiliations:** Chemistry Department, Santosh Bhimrao Patil Arts, Commerce and Science College, Solapur, India

**Keywords:** S-Triazine, semiaromatic polyamides, thermal stability, LOI, solubility

## Abstract

A new aromatic diacid (II) was synthesized and Characterized by Spectroscopic techniques namely, FT-IR, ^1^ H and ^13^ C NMR, etc.

A series of aromatic aliphatic polyamides containing phenoxy s-triazine ring with methylene spacer group was synthesized from diacid (II) and various aromatic diamines by using Yamazaki Phosphorylation method. These polyamides were obtained in good yields and characterized by solubility in common organic solvent, inherent viscosity, FT-IR, X-ray diffraction analysis. All of these polyamides were found to be amorphous in morphology as indicated by XRD to posses outstanding solubilities, and to be easily dissolved in amide-type polar aprotic polar solvents. Polyamides with moderate inherent viscosity in the range 0.21 to 0.41 dL/g in N,N,dimethyl formamide solvent (DMF) at 30 ± 0.1° C. The Thermal properties of the polyamides were evaluated by Thermogravimetric analysis and Differential scanning calorimetery. These polymer shows good thermal stability with glass transition temperature (T_g_) of 143–223°C and their (T_max_) weight loss temperature were around 426–455°C, confirming their good thermal stability. The char yields of these polymers were given their limiting oxygen index LOI 32.3 to 37.5 5% values of polyamides; indicate these polymers also show good flame resistance. The NPs were negatively charged with a zeta potential of −24.2 to −37.9 mV indicating a good colloidal stability against aggregation.

## 1. Introduction

Direct polycondensation under mild conditions is probably the most elegant and valuable method available for obtaining wholly or partly aromatic light molecular weight polyamides since about 1900 to till today, considerable research effort. Aromatic polyamides are thermally stable polymers with a favourable balance of physical and chemical properties. A great deal of effort has been directed into the development of aromatic heterocyclic polymers for use as advanced composite matrices, structural adhesives, or coatings in high-temperature applications [1,2].

All over the world has been devoted to aromatic polyamides because of their special properties. They are thermally stable polymers more or less rod like, depending on the units, spinable in solution to give ultra high strength, high modules fibers, fire resistant and exhibit liquid crystal behavior. They are also very useful material for the military aeronatic and space industries.

In the literature direct polycondensation of aromatic compounds was carried out by N. Yamazaki [3] and Matsumoto in N-methyl pyrrolidone with triphenyl phosphite, pyridine, LiCl, many studies have been undertaken to there as the degree of polycondensation. The direct polycondensation gives low result in both molecular weight and yield when applied to wholly aliphatic monomers mainly for two reasons a) the absence of suitable solvent for highly crystalline aliphatic polyamides, and b) too high basicity of the aliphatic amino groups. Wholly aromatic compounds were partially attributed to the low solubility of polyamides due to amide linkages present shows strongly hydrogen bonding interaction leads more interchain packing.

Therefore, over the past decade, much research has been conducted into the chemical modification of semi-aromatic polyamides (PA)s to improve their processability. Many researchers [4,5] have focused on developing structurally modified PAs by changing the structures of the bulky, packing-disruptive monomers through the introduction of flexible units and bulky side groups and by breaking the regularity and symmetry of the polymer chain. Among the PAs in this category,s-triazine-containing polymers are attracting a great deal of interest due to their excellent thermal resistance and high tensile strengths and moduli at elevated temperatures compared to conventional polymers. The synthesis of triazine polymers have been considerably investigated by different research groups [6–10]. The properties of the s-triazine polymers are influenced by the nature of substituents on the s-triazine nucleus and also by the nature of diamine components of polymer chain. These aromatic polyamides are promising materials of high thermal stability and processibility. The synthetic procedure of the diacid monomers, 2,4-bis-(4-carboxyphenoxy)-6-thiophenoxy-s-triazine and 2,4-bis-(3-carboxyphenoxy)- 6-thiophenoxy-s-triazine, was similar to that of Sagar et al. [11] described in the cardo section of their manuscript. The reaction of the diacids with commercially available aromatic diamines using Yamazaki’s phosphorylation reaction gave the polymers.

Synthesized a new set of high-performance polyamides based on s-triazine ring as flexibilizing linkages into the backbone of wholly aromatic polyamides, which results in soluble polyamides with higher thermal stability along with good processability [12–20].

A series of novel aromatic aliphatic polyamides containing phenoxy s-triazine ring with methylene spacer group was synthesized from a new aromatic diacid namely 2,4-bis-(4-carboxy methylene phenoxy) 6-phenoxy-s-triazine diacid and charactized by spectroscopic techniques namely, FT-IR, C^13^ NMR, ^1^ H NMR etc. Newly synthesized and various commercially available aromatic diamines were subjected to polymerization by using Yamazaki phosphorylation method. These polyamides were obtained in good yields and characterized by solubility in common organic solvents, viscosity, FT-IR, X-ray, diffraction studies and thermogravimetric analysis and LOI. The polyamides had inherent viscosity in the range of 0.21 to 0.41 dL/g in N,N dimethyl formamide (DMF) at 30°C. All the polyamides were readily soluble in polar aprotic solvents such as DMF, N-methyl 2-pyrrolidone (NMP), N,N, dimethyl acetamide, N,N,dimethyl sulphoxide etc. due to their amorphous morphology as indicated by XRD.

## 2. Experimental

### 2.1. Materials

Cyanuric chloride, various aromatic diamines namely, bis-4-(4ʹ-methylene phenylene amine) (MDA), 4,4ʹ-oxy dianiline (ODA), 4,4ʹ-sulphonyl diphenyldiamine (SDA), p-phenylene diamine, m-phenylene diamine,(sigma Aldrich Make), and 4-hydroxy phenyl acetic acid, phenol, (sd-fine make); Hydrazine hydrate (80%) (sd-fine make); Silver nitrare (sigma Aldrich Make), were used as received. N-methyl 2-pyrolidone was dried by azeotropic removal of water with benzene and distilled under reduced pressure and distillate was stored over linde type 4A° molecular sieves.

#### 2.1.1. Measurements

The FT-IR spectra of organic compounds and polymers were recorded using Nicolet spectrometer. ^1^ H NMR (400 MHz) and ^13^ C NMR (100 MHz) spectra were obtained with a Bruker Advance spectrometer at 25°C using CDCl_3_ and DMSO- d_6_ as solvent. Polyamides inherent viscosities were obtained with a polyamide concentration of 0.5 g/dL in DMF solvent at 30°C using an Ubbelhode suspended level viscometer. Differential scanning calorimetry (DSC) was measured on a Mettler Toledo DSC STAR ^e^ instrument at heating rate of 20°C/min. under nitrogen. The glass transition temperatures (T_g_) were determined from DSC curves. Thermogravimetric analysis (TGA) was performed on a Mettler Toledo STAR ^e^ instrument at a heating rate of 10°C/min under nitrogen. Wide angle X-ray diffraction (WAXD) was measured with a Rigaku X-ray diffractometer using polyamide powder.

### 2.2. Diacid synthesis

#### 2.2.1. Synthesis of 2,4-dichloro 6-phenoxy s-triazine (I)

In a one- neck 100 mL round bottom flask equipped with magnetic stirrer, dropping funnel,were added 7.2 g (0.04 mol) cyanuric chloride, 50 mL dichloro methane. By keeping reaction mixture in ice bath and maintaining temperature between 0 and 5°C, were added the solution 3.6 g (0.04 mol) phenol and 1.6 g (0.04 mol) NaOH in 20 mL water were added dropwise over a period of 15 min. Reaction mixture was stirred for 3 hr at room temperature. After the completion of reaction organic layer was separated from aqueous layer. Organic layer was washed with 10% aq. NaOH solution to remove unreacted phenol; if any. The organic layer was washed with distilled water, dried over anhydrous Na_2_SO_4_, filtered and then solvent was removed under reduced pressure. The white solid product was recrystalized from the mixture of hexane and chloroform (3:1). The yield; 8.2 g (88%), M.p. 111–112°C.

#### 2.2.2. Synthesis of diacid 2,4-Bis-(4ʹ-carboxy methylene phenoxy)-6-phenoxy-s-triazine (II)

A one neck 100 mL round bottom flask was placed 4.82 g (0.02 mol) 4-dichloro 6-phenoxy s-triazine (I), 50 mL dichloromethane and reaction mixture was cooled in ice bath. In another beaker a mixture of 4-hydroxy phenyl acetic acid 6.64 g (0.04 mol), 3.6 g (0.09 mol), of sodium hydroxide in 30 mL distilled water was prepared and then it was added to the reaction mixture through dropping funnel maintaining temperature 0–5°C. The reaction mixture stirred for 3 hr at room temperature; poured the reaction mixture into the acidified water to precipitate the solid. It was filterated on Buchner funnel, washed with hot water, dried under vacuum pump at 100°C. Yield 9 g, (90%), M.p. 230°C.

FT-IR (KBr,cm^−1^): 2600–3300 (-OH of – COOH), 1712 (-CO stretching of – COOH),

1214 (-O-), 1500,1574 (-C-N and – C = N).

^1^ H NMR (in ppm, ∂) 7.02 (m,5 H), 7.08 (dd,1 H), 7.22 (d,2 H), 7.325 (d,2 H), 7.694 (s,1 H).

### 2.3. Polymerization

#### 2.3.1. Synthesis of polyamide PA-1

To a dry three neck round bottom flask (100 mL) equipped with magnetic stirrer, condenser, nitrogen gas inlet; were added 1 mmol of aromatic diacid (II) 0.473 g and 4,4-oxy dianiline (ODA) 1 mmol i.e., 0.200 g, 0.200 g lithium chloride (8 wt% based on solvent N-methyl pyrrolidone (NMP) and pyridine mixture.) 0.744 g (0.63 mL 2.4 mmol) triphenyl phosphite, 0.5 mL pyridine and 2 mL NMP. The mixture was stirred well and temperature was slowly raised to 100°C over period of half an hour. Reaction mixture was stirred at 100°C for 3 h. After cooling the resultant viscous solution was poured into rapidly stirred 300 mL methanol; when polymer precipitates out. Polyamides was filtered, washed with hot water, then methanol and dried at 100°C for 8 h. The series of PA-1 to PA-5 aromatic polyamides was synthesized by above procedure.

#### 2.3.2. Preparation of silver nanoparticles using PA-1 to PA-5 polymer

Silver nanoparticles have been extensively studied in the field of nanotechnology due to its broad range of applications. Different studies focused on immobilization of silver nanoparticles on different substrate; silica is one of the most studied support for silver nanoparticles immobilization, because of its high surface area, low cost, and ease of functionalization for different substrates. In present study, we used in a typical experiment, different volumes of hydrazine hydrate 80% (0, 10, 20, 50, 100 mL) was immediately added into 50 mL of an aqueous suspension of PA-1 (50 mg), followed by adding of 50 mL of 0.1 M AgNO3 aqueous solution, as summarized in Table 4 . Similarly other polyamides PA-2 to PA-5 were synthesized as per the procedure. The silver colloids were generated during the dropwise addition of silver nitrate solution into suspension solution BDMA in the presence and absence of different hydrazine hydrate volume (0, 10, 20, 50, and 100 mL) within 5 min. The solution was centrifuged at 12,000 rpm/min for 10 min and the sediment washed with distilled water and methanol 4 times.

**Table 1.** Inherent viscosity, yield of ^a^polyamides from ^b^diacid (II) and various aromatic diamines

Sr. No.	Polymer code	Aromatic diamine	Yield (%)	^c^Inherent Viscosity,η_inh, (dL/g)_
1.	PA-1	ODA	95	0.41
2.	PA-2	MDA	96	0.37
3.	PA-3	SDA	95	0.34
4.	PA-4	p-PDA	97	0.28
5.	PA-5	m-PDA	98	0.21

a) Polymerization was carried out with 1 mmol each of aromatic diacid (II) and various aromatic diamine.

b) 2, 4-bis-(4-carboxy methylene phenoxy)- 6-phenoxy-s-triazine (II).

c) Measured with 0.5% (w/v) polymer solution in DMF at 30 ± 0.1°C.

**Table 2.** Solubility of polyamides PA-1 to PA-5

Polymer	PA-1	PA-2	PA-3	PA-4	PA-5
Solvent					
NMP	++	++	++	++	++
DMF	++	++	++	++	++
DMAc	++	++	++	++	++
DMSO	++	++	++	++	++
Chloroform	–	–	–	–	–
DCM	–	–	–	–	–
THF	–	–	–	–	–

(++) Soluble at room temperature, (–) insoluble.

**Table 3.** ^a^ Thermal properties of polyamides from diacid (XVI) and Various aromatic diamines

Polyamide	^b^ T_g_ (^o^C)	^c^T_i_ (^o^C)	^d^T_10_ (^o^C)	^e^T_max_ (^o^C)	Residual wt.(%)at 900° C	LOI (%)
PA-1	223	250	270	427	37	32.3
PA-2	188	260	296	455	42	34.3
PA-3	196	270	270	461	36	31.9
PA-4	200	265	286	447	43	34.7
PA-5	149	280	291	426	50	37.5

a) Thermogravimetric analysis at a heating rate of 10°C/min. in nitrogen

b) Glass transition temperature, determined by Differential Scanning Calorimeter

(DSC) at a heating rate of 20°C/min. in nitrogen

c) Initial decomposition temperature.

d) Temperature at which 10% wt.loss was observed.

e) Temperature at which maximum rate of degradation was observed.

**Table 4.** Measurement of Zeta potential by coating of silver nano particle with hydrazine hydrate

Sr. No.	Polymer code	Hydrazine hydrate (80%) (mL)	Zeta potential (mv)
1.	PA-1	0	−10.42
2.	PA-2	10	−2.56
3.	PA-3	20	−2.20
4.	PA-4	50	−3.69
5.	PA-5	100	−12.87

### 2.4. Result and Discussions

**Scheme 1.**
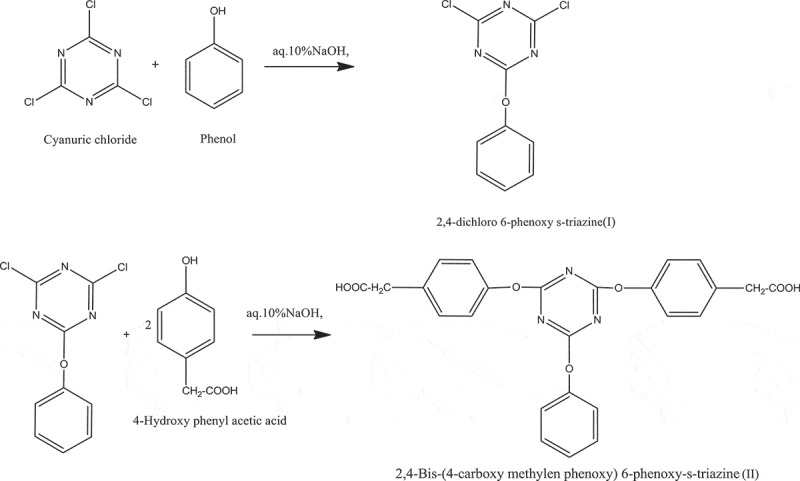
Synthesis of 2,4-Bis-(4ʹ-carboxy methylene phenoxy)-6-phenoxy-s-triazine(II)

The aromatic polyamides are the high-performance polymers. To improve the processability of these polymers in the present studies, incorporation of flexible moiety as well as pendant phenoxy moiety was introduced successfully. Thus we have incorporated aromatic thermally stable 1,3,5-s-triazine moiety containing pendant phenoxy group and methylene spacers so as to improve the solubility of polymers. We have synthesized new aromatic diacid 2,4-bis-(4-carboxy methylene phenoxy)-6-phenoxy-s-triazine (Scheme 1). The first chlorine atom of cyanuric chloride was replaced with phenol at low temperature to form 2, 4-dichloro-6- phenoxy-1,3,5-s-triazine. The remaining two chlorine atoms of cyanuric chloride were then replaced via.substitution reactions of 4-hydroxy phenyl acetic acid with 2,4-dichloro-6- phenoxy-1,3,5-triazine to give the s-triazine (Scheme 1). The s-triazine unit containing new diacid (II) was characterized by different spectral techniques.

#### 2.4.1. Diacid synthesis

The aromatic aliphatic diacid 2,4-bis-(4-carboxy methylene phenoxy)-6-phenoxy-s-triazine (XVI) was synthesized from cyanuric chloride and phenol and 4-hydroxyphenyl acetic acid and characterized by FT-IR, NMR (^1^ H,^13^ C).

#### 2.4.2. Charactrization of monomer

**Figure 1.**
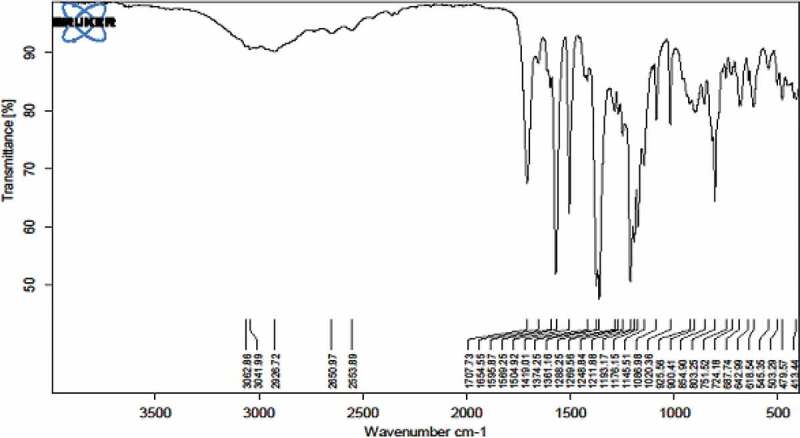
FT-IR spectrum 2,4-Bis-(4ʹ-carboxy methylene phenoxy)-6-phenoxy-s-triazine (II)

The structure of 2,4-bis-(4-carboxy methylene phenoxy) 6-phenoxy-s-triazine was confirmed by FT-IR spectrum (Figure 1). It showed characteristic absorption bands at 3042 (-OH stretching of carboxylic acid), 1712 (-CO-stretching of carboxylic acid), 2860 (aliphatic-CH), 1214 (-O- ether stretching), 1500 and 1574 cm^−1^ (-C-N, -C = N, stretching).

**Figure 2.**
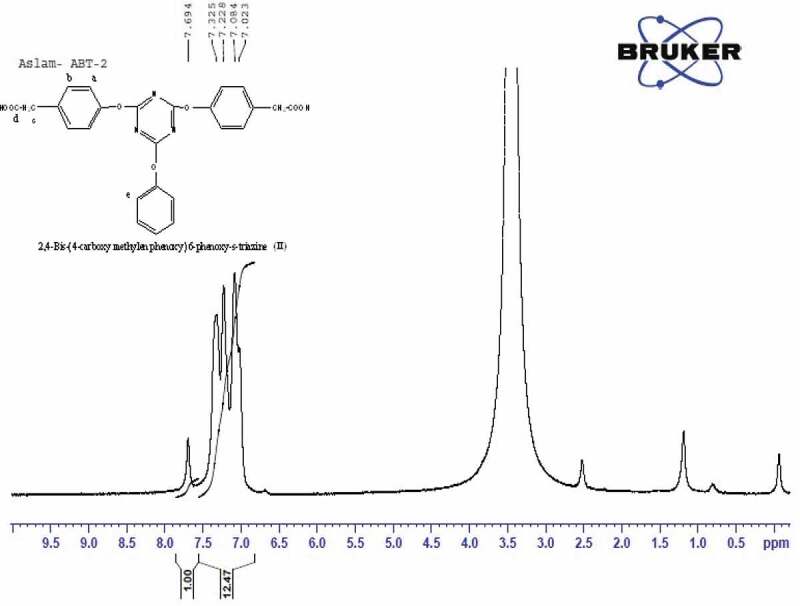
^1^ H NMR spectrum2,4-Bis-(4ʹ-carboxy methylene phenoxy)-6-phenoxy-s-triazine(II)

The ^1^ H NMR (Figure 2) spectrum of (II) showed signals (in ppm, ∂) at 4.0 (s, 2 H, H_c_), 7.02 (m,5 H, H_e_),7.08 (dd,1 H, H_a_),7.325 (d,2 H, H_b_), and carboxylic acid proton at 7.694 (s,1 H).

**Figure 3.**
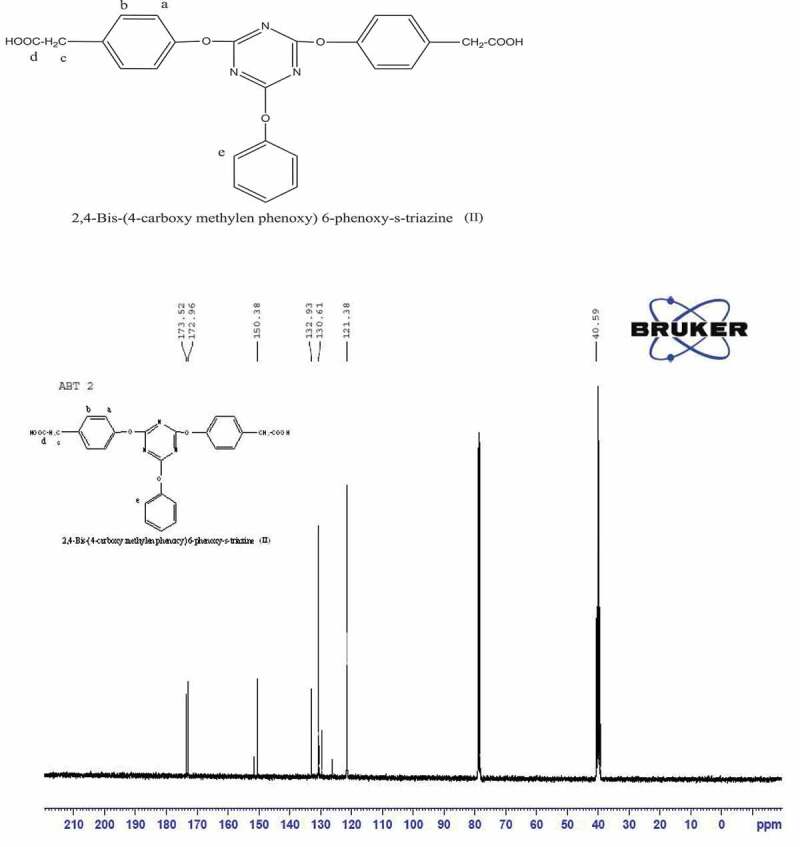
^13^ C NMR spectrum of2, 4-Bis-(4ʹ-carboxy methylene phenoxy)-6-phenoxy-s-triazine(II)

**Figure 4.**
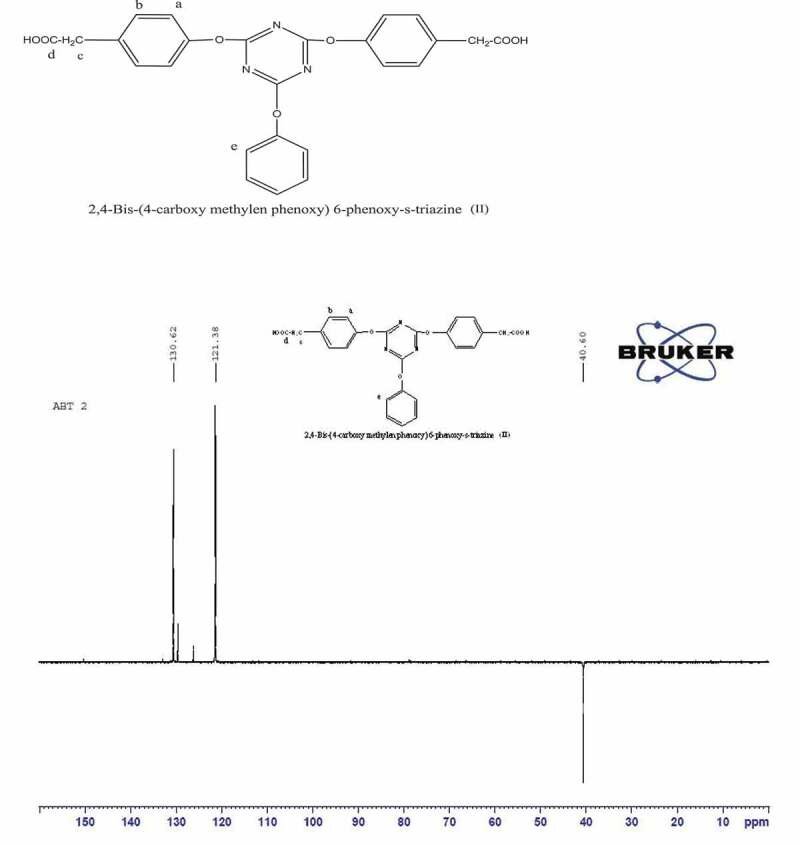
DEPT ^13^ C NMR spectrum of 2,4-Bis-(4ʹ-carboxy methylene phenoxy)-6-phenoxy-s-triazine (II)

The ^13^ C NMR and DEPT ^13^ C NMR spectra (Figures 3,Figures 4) show various ^13^ C peaks. Aliphatic C signal appeared at 40 ppm. Carbon attached to oxygen showed signal at 150 ppm, while other aromatic carbons signal at 121–130 ppm were observed. DEPT ^13^ C NMR spectrum confirmed CH_2_ peak at 40 ppm which appeared downword; CH appeared upword where as tertiary carbon signals disappeared.

**Figure 5.**
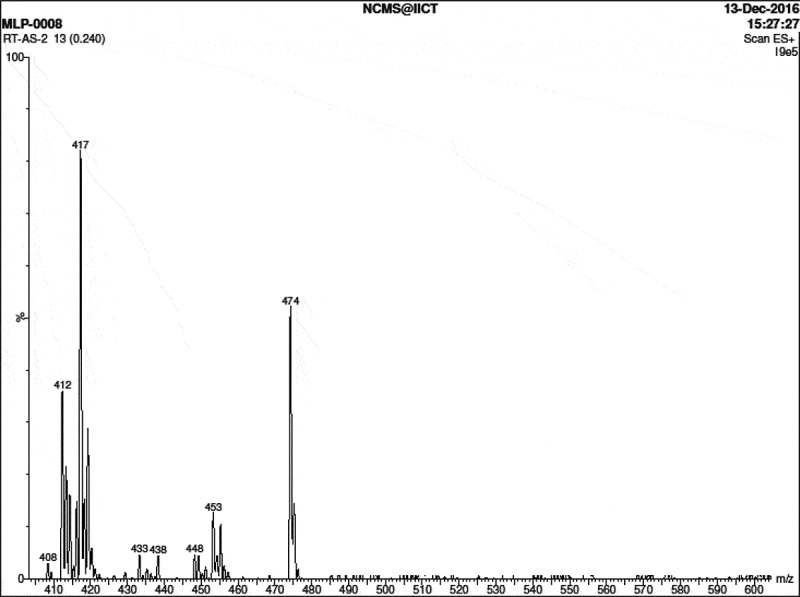
Mass spectrum of2,4-Bis-(4ʹ-carboxy methylene phenoxy)-6-phenoxy-s-triazine (II)

Mass spectrum of (II) in (Figure 5) showed (M + 1) peak as strong base peak at 474 indicating molecular formula weight of 473 for diacid (II), corresponding to molecular formula; C_25_ H_19_N_3_O_7_.

## 3. Polymer synthesis

**Scheme 2.**
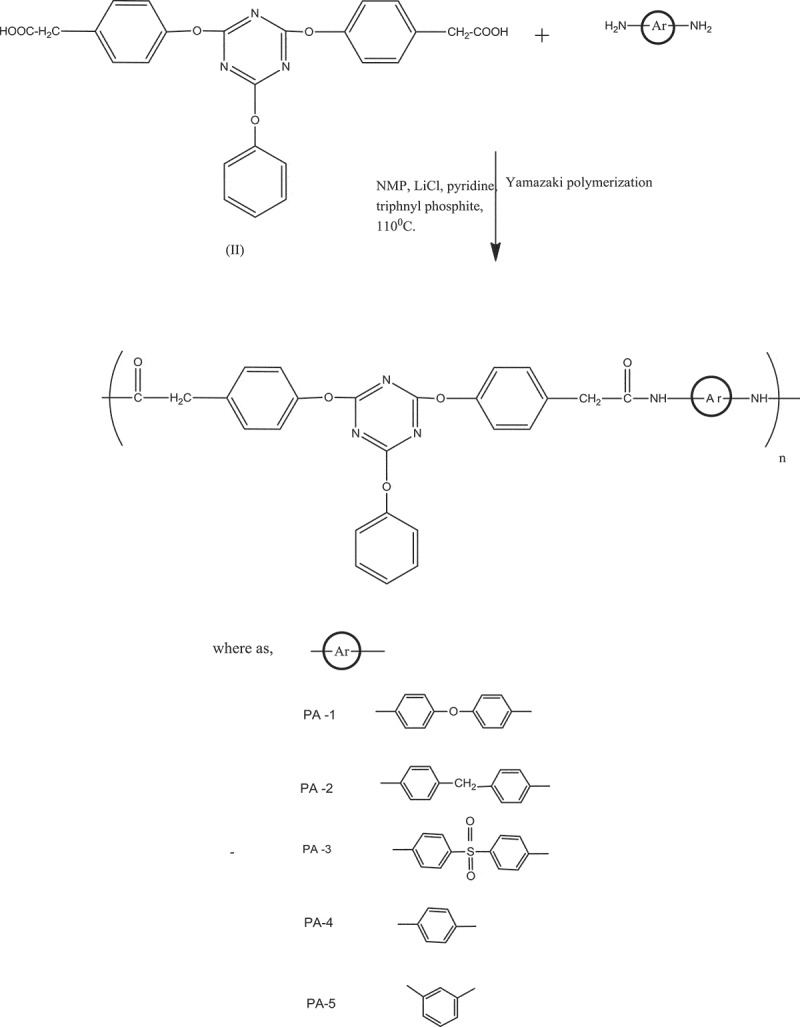
Synthesis of Polyamides from diacid (II) and various aromatic diamines

The polyamides were synthesized from 2, 4-bis-(4-carboxy merthylene phenoxy)-6-phenoxy-s-triazine and various aromatic diamines by Yamazaki phosphorylation polymerization method. (Scheme 2.)

These polyamides showed inherent viscosities in the range of 0.21 to 0.41 dL/g, indicating build up of moderate molecular weights.

### 3.1. Polymer characterization

**Figure 6.**
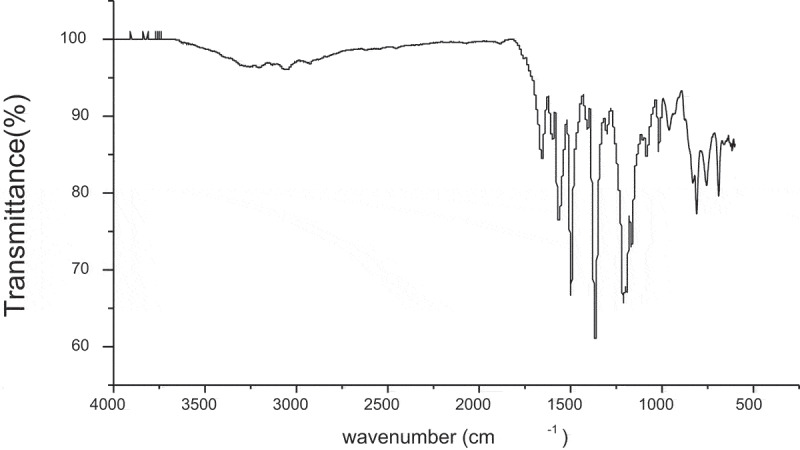
FT-IR spectrum of polyamide (PA-1)

The structures of polymers were characterized by FT-IR spectroscopy. A FT-IR spectrum (cm^−1^) of PA-1 showed (Figure 6) absorption bands at 3300 (-NH stretching), 3039, (aromatic -CH), 2937 (aliphatic – CH stretching), the sharp bands at 1670–1650 (amide I) and 1530–1520 (amide II).

### 3.2. Polymer solubility

The solubility behavior of these polyamides series PA-1 to PA-5 was tested in various organic solvents and the results are summarized in Table 1–2. Because of the introduction of heterocyclic 1,3,5-s-triazine with pendant phenoxy group and aryl ether linkage and methylene spacer groups in structure of aromatic diacid, all the resulting polyamides were readily soluble in polar solvents such as DMSO, NMP, DMF, DMA_C_, etc. The good solubility makes these processable polymers important for industrial applications. The incorporation of nonlinear chain segments say m-catenation, into a PA tends to significantly improve its solubility.

### 3.3. Thermal properties

**Figure 7.**
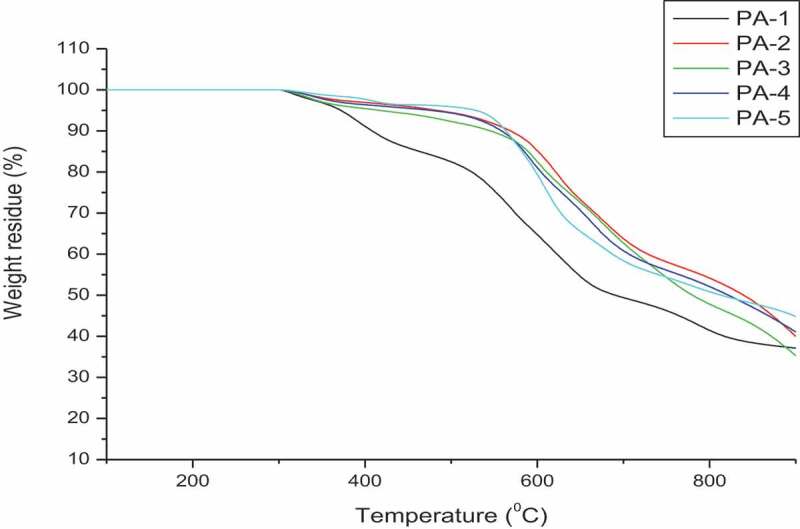
TGA curves of Polyamides PA-1 to PA-5

Thermal stability of polyamides was determined by thermogravimetric analysis. Typical TGA curves of polymer under nitrogen atmosphere at the heating rate of 10°C/min; is shown in (Figure 7). Initial decomposition temperature T_i_ and temperature at 10% weight loss (T_10_) were determined from thermograms and the data is given in (Table 3), T_i_ and T_10_ values are in the range 250–280°C and 270–296°C, respectively. The temperature at which maximum weight loss (T_max_) was observed for these polyamides is in the range of 426 to 461°C indicating the good thermal stability of polymers. The char yields of these polyamides at 900°C were in the range of 37 to 50%.

**Figure 8.**
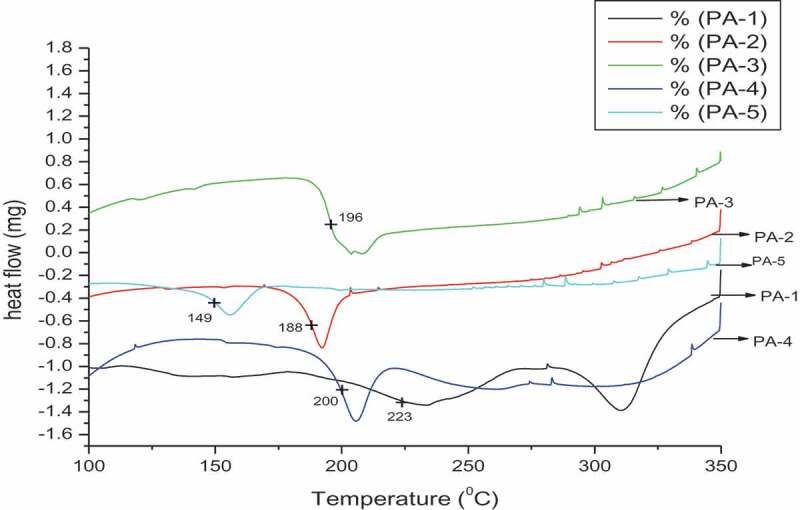
DSC curves of polyamides PA-1 to PA-5

DSC was used to evaluate the thermal transitions of these polyamides. Glass transition temperatures (T_g_) were determined under nitrogen at heating rate 20°C/min. The DSC curves are shown in (Figure 8) and results are summarized in Table 3. T_g_ were in the range of 149 to 223°C, low (T_g_) 149°C of PA-15 due to m-catenation i.e., 1,3-position of aryl amide group leading to kink and irregular arrangement of constituents thus preventing the close packing structure of polyamides; there by allowing more mobility. While 1,4- position of aryl amide group shows rigidity of polymer (PA-14) material due to strong interaction between hydrogen bonding in amides and regular close packing structure, thus raising T_g_ to 200°C. Theoretical value of limited oxygen index (LOI) was calculated using the char residue (CR) residue employing Van Krevelen correlation [21]: LOI = 17.5 + 0.4(CR). LOI is defined as the minimum amount of oxygen (in percentage) that will sustain the combustion. Polymers exhibiting values larger than 26 are considered to be flame retardants [22,23]. The NPs were negatively charged with a zeta potential of −24.2 to −37.9 mV indicating a good colloidal stability against aggregation [24,25]. Our main approach is use of synthesized s-Triazine based polyamide as substrate for nanoparticle materials.

**Figure 9.**
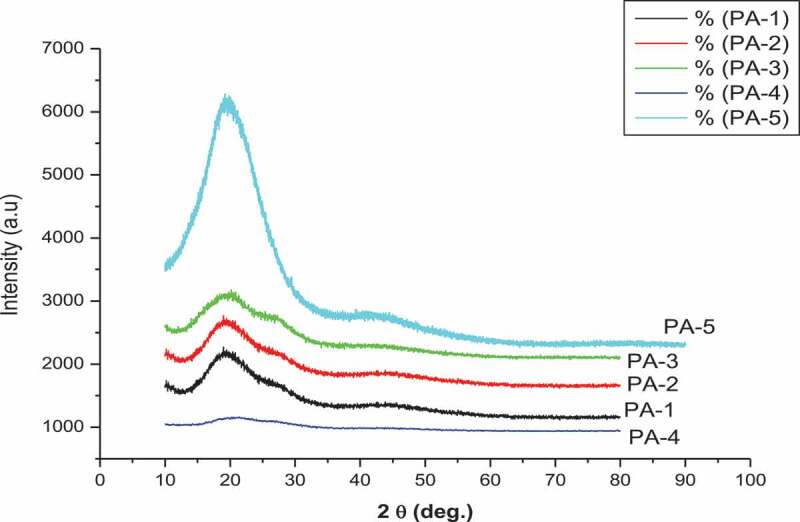
XRD curves of polyamides from PA-1 to PA-5

**Figure 10.**
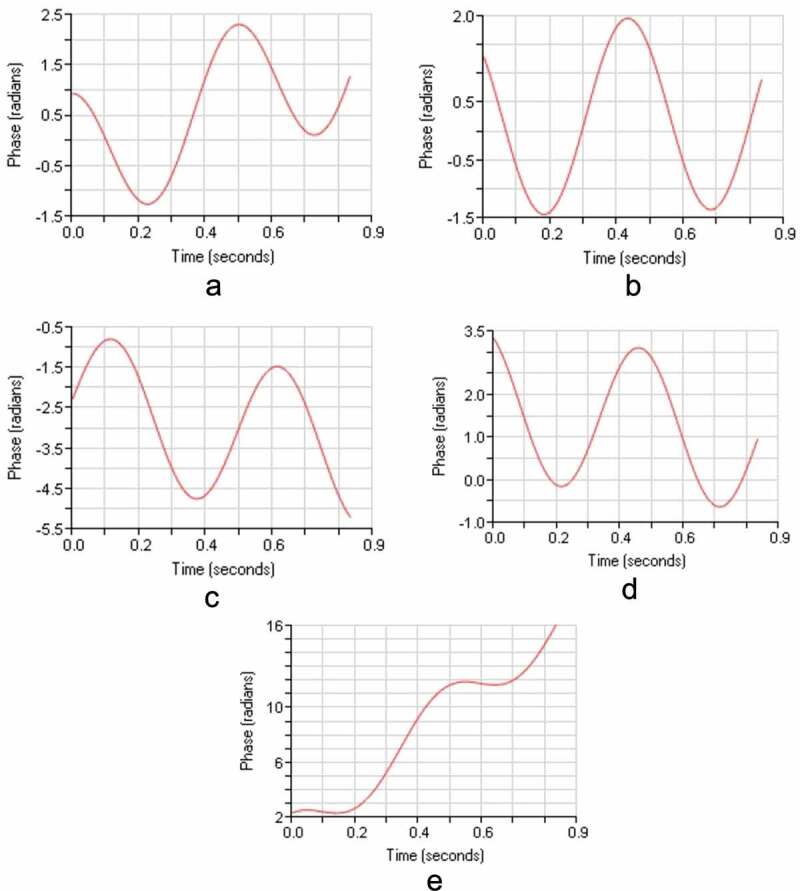
Zeta potential for polyamides

### 3. 4. X-ray diffraction

In order to study the crystalline or amorphous nature of the polyamides WAXD measurement at room temperature were performed. Typical wide-angle X-ray diffractograms of the powder polyamids are illustrated in (Figure 9–10) WAXD analysis of polyamides revealed the polyamides were amorphous in nature showing broad peaks in the region 2θ = 15 to 30° due to flexible linkages (–O – and – SO_2_–) in the main chain of the polymer, which reduce interactions among chains and result in poorer chain packing, leading to amorphous PAs with improved solubility and processability. This agrees with the general rule that solubility decreases with increasing crystalinity.

### 3.5. Zeta potential

As per the procedure of coating silver nanoparticle by addition of various volume of hydrazine hydrate (80%) summarized in Table 4. Indicates that zeta potential is negative value which imparts the immobilization nature of silver nanoparticle on the surface of synthesized polyamide based on s-triazine moiety and methylene spacer group. The various graphical representation of zeta potential as shown in figures below.

Fig. a. Zeta potential PA-1

Fig. b. Zeta potential PA-2

Fig. c. Zeta potential PA-3

Fig. d. Zeta potential PA-4

Fig. e. Zeta potential PA-5

## 4. Summary and conclusion

The synthesized aromatic polyamide based on s-Triazine moiety and methylene spacer group which showed good solubility property without affecting thermal stability. These polyamide exhibit good flame resistance property by showing LOI more than 26%. Polyamide coated with silver nanoparticle exhibit colloidal affect and immobilization property. The silver nanoparticle coated polymer useful for adherent of different substrate due to its immobilization property. These polyamide containing s-triazine and methylene spacer group showed symmetrical behaviour with improve processability and thermal stability by incorporation of heterocyclic ring into the backbone of polymer.

Main approach is synthesis of Polyamide based on symmetrical unit of heterocycle s-Triazine moiety and methylene spacer group which improve the processabiity without much more affect thermal stability. The flexibility of polyamides which were measured by Glass transition temperature become less than 200°C improved by methylene spacer group. The processable polyamides based on these structural modification important for silver nanoparticle which shows immobilization as collidal property.
